# Sequential invasions by fruit flies (Diptera: Tephritidae) in Pacific and Indian Ocean islands: A systematic review

**DOI:** 10.1002/ece3.8880

**Published:** 2022-04-30

**Authors:** Pierre‐François Duyck, Hervé Jourdan, Christian Mille

**Affiliations:** ^1^ CIRAD, UMR PVBMT Noumea New Caledonia; ^2^ IAC, Equipe ARBOREAL La Foa New Caledonia; ^3^ Aix Marseille Univ, Avignon Univ, CNRS, IRD, IMBE Noumea New Caledonia

**Keywords:** biosecurity, exotic species, human‐mediated dispersal, interspecific competition, patterns of invasion

## Abstract

The aim of our review was to examine the cases of Tephritidae invasions across island systems in order to determine whether they follow a hierarchical mode of invasion. We reviewed the literature on factors and mechanisms driving invasion sequences in Pacific and Southwest Indian Ocean islands and gathered every record of invasion by a polyphagous tephritid in island groups. From invasion date or period, we defined an invasion link when a new fruit fly established on an island where another polyphagous tephritid is already resident (that was indigenous or a previous invader). Across surveyed islands, we documented 67 invasion links, involving 24 tephritid species. All invasion links were directional, i.e., they involved a series of invasions by invaders that were closely related to a resident species but were increasingly more competitive. These sequential establishments of species are driven by interspecific competition between resident and exotic species but are also influenced by history, routes, and flows of commercial exchanges and the bridgehead effect. This information should be used to improve biosecurity measures. Interactions between trade flow, invasive routes, and the presence of invasive and resident species should be integrated into large‐scale studies.

## Graphical abstract

We gathered observations of invasions by polyphagous tephritids in islands. All invasion links were directional, i.e., they involved a series of invasions by invaders that were closely related to a resident species but were increasingly more competitive. These sequential establishments of species are driven by interspecific competition between resident and exotic species but are also influenced by history, routes, and flows of commercial exchanges and the bridgehead effect.

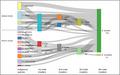

## 1 INTRODUCTION

Globally, the increase in the number of invasive alien species shows no sign of saturation, with an increasing number of species threatening to invade new territories (Seebens et al., 2017). The threat of invasion is especially high in remote places such as isolated islands because they often offer vacant niches that alien species can reach via new human‐created pathways (Moser et al., 2018; Seebens et al., 2018). Communities that are vulnerable to invasion have unsaturated niche space mainly because of evolutionary history in isolation (islands), dispersal limitation, and/or anthropogenic disturbance (David et al., 2017).

Understanding general rules governing invasions is required to improve biosecurity measures and therefore to develop sustainable agriculture and to conserve biodiversity (Hulme, 2010; Sikes et al., 2018). Indeed, if many pests are already causing significant damage to crops in various territories, the most serious in terms of crop damage and effects on human populations are often those which are not yet present but which threaten to invade (Fournier et al., 2019; Hulme, 2021; Paini et al., 2016). Once an exotic species has been established in a territory, its eradication is often impossible, and the implementation of control measures is difficult, expensive, and often polluting (Simberloff et al., 2013).

Many species in the family Tephritidae (true fruit flies) are major pests of fruit and vegetable crops in most tropical and subtropical countries (Qin et al., 2015; Schutze et al., 2015; Vargas et al., 2015; White & Elson‐Harris, 1992). Numerous cases of biological invasions have been observed in this family around the world despite serious and strong quarantine procedures (Clarke et al., 2005; Duyck et al., 2004; Ekesi et al., 2009; Moquet et al., 2021). Most of these invasions have catastrophic consequences, including the destruction of fruits and vegetables and even the destruction of entire crops, dependence on imports, change of the phytosanitary status in the context of the International Standards for Phytosanitary Measures and the International Plant Protection Convention (IPPC) (https://www.ippc.int/) resulting in the inability to export when a quarantine regulated species arrives, and the use of chemicals for control (N’depo et al., 2015). It is therefore essential to avoid and prevent these introductions by strengthening biosecurity procedures (Phillips, 2013).

When introduced, a species may persist only if it is able to pass through environmental and biotic filters (David et al., 2017). Biotic filters include the level and availability of resources, competition, and natural enemies, which define the realized niche (Broennimann et al., 2007). Because invasive species must exploit available resources in the recipient ecosystem, they establish trophic interactions with the resident species (David et al., 2017). But, indirect biotic factors such as exploitative competition, while they often occur in invasion processes (White et al., 2006), are rarely studied in detail because assessing resource density and dynamics is difficult (Hart et al., 2018).

In the Tephritidae family, abundance and distribution are mainly structured by both abiotic factors, such as temperature and humidity, and biotic ones, mostly host plant distribution and abundance (Duyck et al., 2004; Facon et al., 2021). Although interspecific competition among Tephritidae may not be very important in native communities (Clarke, 2017), in an unstable situation, such as occurs when resident species interact with closely related invasive species, this interspecific exploitative competition may be very strong and asymmetrical, leading to the competitive exclusion or displacement of resident species (Duyck et al., 2004, 2006; Ekesi et al., 2009; Moquet et al., 2021). In contrast, substantial interspecific competition at the inception of invasion may prevent the establishment of less competitive species. This should subsequently result in invasion sequences that involve increasingly competitive invasive species. In their review, Duyck et al. (2004) listed cases of invasions by polyphagous Tephritidae species and showed that these invasions were “sequential”, i.e., they involved a hierarchical mode of invasion with invaders being increasingly competitive over time. Trade history and networks also affect the distribution and occurrence of invasive species (Chapman et al., 2017; MacLachlan et al., 2021) and may also lead to a hierarchical mode of invasion (Duyck et al., 2004).

Eighteen years after the synthesis by Duyck et al. (2004), many new cases of invasions by Tephritidae have been documented worldwide. Numerous recent studies on invasive tephritids, whether focused on field data, population genetics, or laboratory experiments (Charlery de la Masselière, Facon, et al., 2017; Clarke et al., 2005; Hafsi, Facon, et al., 2016), have increased our understanding of the invasion process. In particular, *Bactrocera dorsalis* has continued its range of expansion across Africa and in Indian Ocean islands (Ekesi et al., 2016; Hassani et al., 2016; Moquet et al., 2021; Rasolofoarivao et al., 2021).

The aim of our paper is to document invasions of Tephritidae across both Pacific and Southwest Indian ocean islands. We focused on these regions because such island systems offer an ideal opportunity to track and monitor community processes in the context where numerous invasions by tephritids have occurred and have been deeply studied both recently and in the last century with fruit flies. We chose to study invasions on islands because they can be considered to be independent of invasions of continental areas, for which it would be necessary to think of invasions in terms of continuous expansion in space (Moser et al., 2018; Russell et al., 2017). We used previously published data to answer the following questions: (i) Do Tephritidae invasions always follow a hierarchical mode of invasion? and (ii) What are the factors and mechanisms driving invasion sequences?

## 2 METHODS

We conducted a systematic review of literature on polyphagous tephritid invasions in island groups from the Indian Ocean and Pacific region. We first explored published work from Web of Science using the query “Tephritidae AND (invasion OR introduction)” as keywords for islands from Pacific Islands or Southwest Indian Ocean Islands. However, as a lot of information on occurrence and invasion by Tephritidae are present in gray literature, we therefore tracked information from unformal and gray literature such as various proceedings of conferences that took places in the Indian Ocean and Pacific islands and information from leaflets from the Pacific Fruit Fly Project (Land and resource Division, Pacific Community). We also extracted information from PhD theses conducted on Tephritidae in the different islands and older technical reports being in French or English that have been produced by local organizations and services or regional organizations.

The cases selected in the present study need to match different eligibility criteria: (i) the invasive species need to have invaded an island in the Pacific or Indian Ocean, (ii) the invasive species must be a polyphagous tephritid, and (iii) another polyphagous tephritid was already present in the invaded area during the invasion process. These criteria were selected in order to ensure that the ecological niches of the two species largely overlapped. We screened full‐text for the mention of invasion of a polyphagous tephritid and/or for the presence of polyphagous tephritid, being indigenous or previously established, in each reference. Then, we kept all observations of a polyphagous tephritid become established on islands where important polyphagous species were already dwelling because they were indigenous or had previously invaded.

Regarding the coverage of our data set, we need to precise various points regarding both taxonomy and post‐invasion event (such as successful eradication program). Some invasive species, such as *B*. *dorsalis* in Australia or Nauru (Allwood et al., 2002; Suckling et al., 2016), were eradicated after their introduction but have been considered in this review as successful invasions in the presence of one or several already present species. *Bactrocera philippinensis* has been described as invasive in Palau but has been further synonymised with *B*. *carambolae*, which has been synonymised with *B*. *dorsalis* (Clarke et al., 2005; Schutze et al., 2015). Although *B*. *occipitalis* belongs to the *B*. *dorsalis* complex, it is considered a distinct species. *Bactrocera dorsalis* was first identified as *B*. *papayae* in PNG and *B*. *papayae* was later synonymised with *B*. *dorsalis* (Schutze et al., 2015).

Even if it is still dwelling in La Réunion Island, the Mascarene fruit fly *Ceratitis catoirii* is considered to be extinct in Mauritius because it has not been observed since 1957 (White et al., 2006). We therefore have not identified links between this species and species that invaded Mauritius after this date. *Ceratitis malgassa* is considered an endemic species in Mauritius and Comoros (De Meyer et al., 2012), but only a few specimens were observed in the latter study, and the species is rarely mentioned in the literature. We are not sure whether this species was present in Mauritius before the first species invaded. We therefore did not include this species in defining invasion links for these territories. Regardless, these cases would not modify invasion sequences as these species are not invasive in other territories.

For most of the studied islands, dates or periods of invasion by polyphagous Tephritidae have been documented (Appendix S1), but one limitation on this aspect of our study could be the accuracy about the date of invasion. A lag of detection may exist according to the presence and extent of a monitoring network. However, we considered that invasion by polyphagous Tephritidae is usually so dramatic for fruit producers that invasion and spread of new species are quickly detected even in the context of lack of regular monitoring. When no date was available or in case of uncertainty, we used a range of the date of invasion.

We defined an “invasion link” as occurring when a species established on an island in the presence of another polyphagous tephritid (indigenous or previously established). Several invasion links are established for islands where several polyphagous tephritid were present when the invasion occurred. When several species subsequently invaded the same island, we defined this situation as an “invasive series”.

In order to visualize invasion links, we performed a diagram using the Sankey Network function from package *networkD3* (Allaire et al., 2017) in R statistical software v4.0.2 (R Core Team, 2021) with the different Tephritidae species set as nodes and the invasions links between two species as links using the number of invasions links recorded between two species as value.

## 3 RESULTS AND DISCUSSION

### 3.1 Invasions links are directional

**FIGURE 1:**
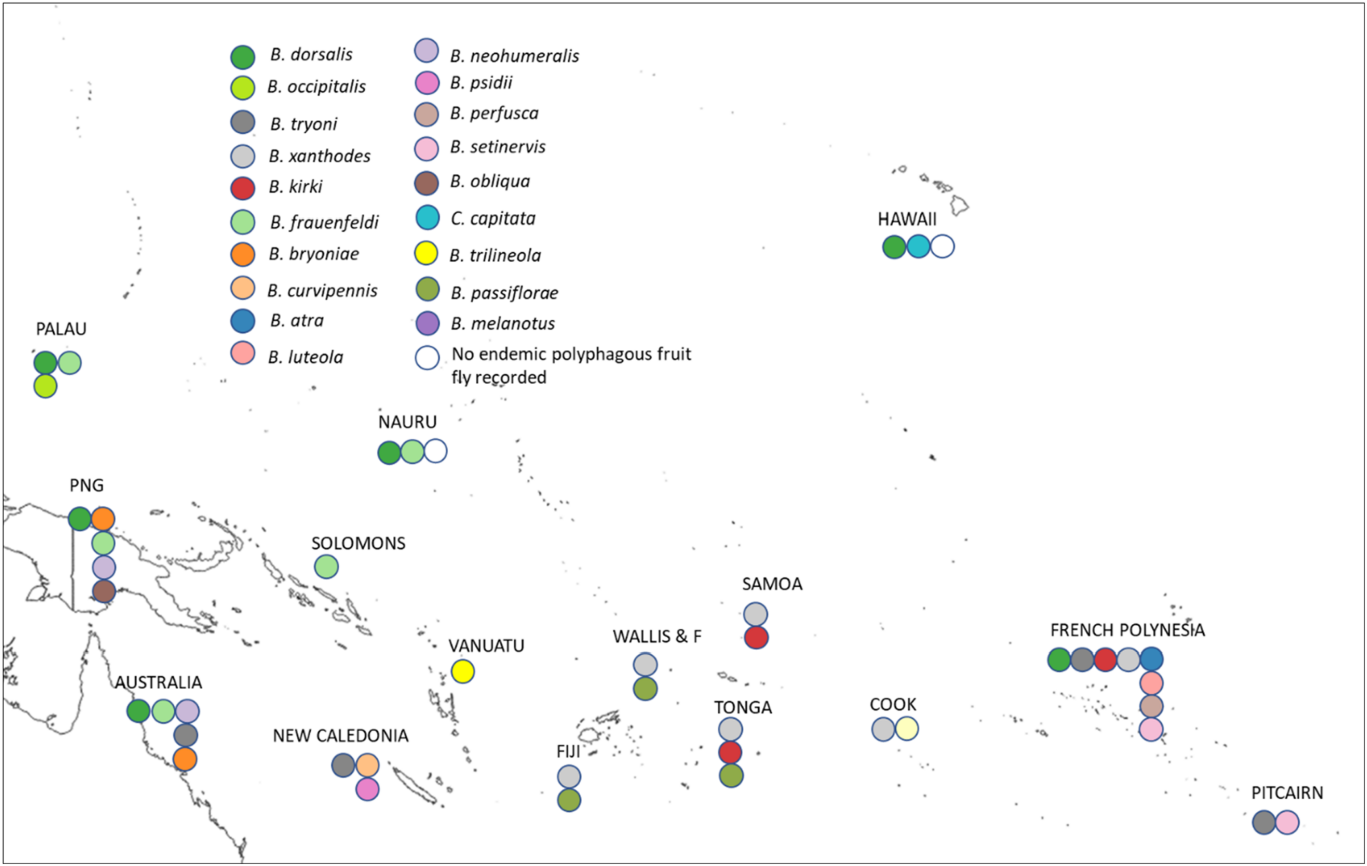
Invasion among 15 Pacific territories by polyphagous fruit flies. For each territory, the sequence of circles extends from indigenous species (when present) on the right to invasive species to the left, with 1st‐, 2nd‐, 3rd‐, and 4th‐order invaders in temporal sequence from right to left using invasion date or period from Appendix S1. Some species were eradicated after introduction (such as *B*. *dorsalis* in Nauru and in Australia) but represent successful cases of invasion of one polyphagous species in the presence of another polyphagous species

**FIGURE 2:**
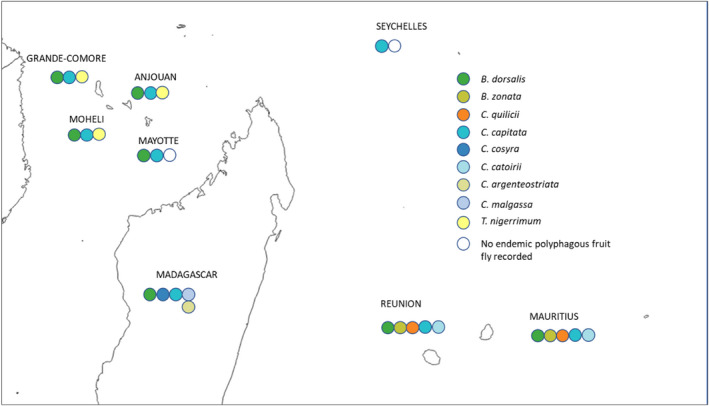
Invasion of eight islands in the Southwest Indian Ocean by polyphagous fruit flies. For each territory, the sequence of circles extends from indigenous species (when present) on the right to invasive species to the left, with 1st‐, 2nd‐, 3rd‐, and 4th‐order invaders in temporal sequence from right to left using invasion date or period from Appendix S1

Based on our extensive literature review, we documented 67 invasion links of an invasive polyphagous tephritid species occurring with a resident polyphagous tephritid species, i.e., an indigenous species or a previous invader that achieved establishment in Pacific and Southwest Indian Ocean islands (Appendix S1, Figures 1 and 2). Our study focuses on 24 fruit species, which are involved in at least one invasion situation (Figures 1 and 2). All invasion links documented in our review were directional: reciprocal invasions were never documented, i.e., if species A invaded an island occupied by species B, then B never invaded an island previously occupied by A. These invasion links were also “transitive” (i.e., when A invaded in the presence of B, and B in the presence of C, there are some instances of A invading in the presence of C but not the reverse). Thirty links of invasions have been documented in the Pacific islands, and thirty‐seven links of invasions have been documented in the Southwest Indian Ocean islands.

**FIGURE 3:**
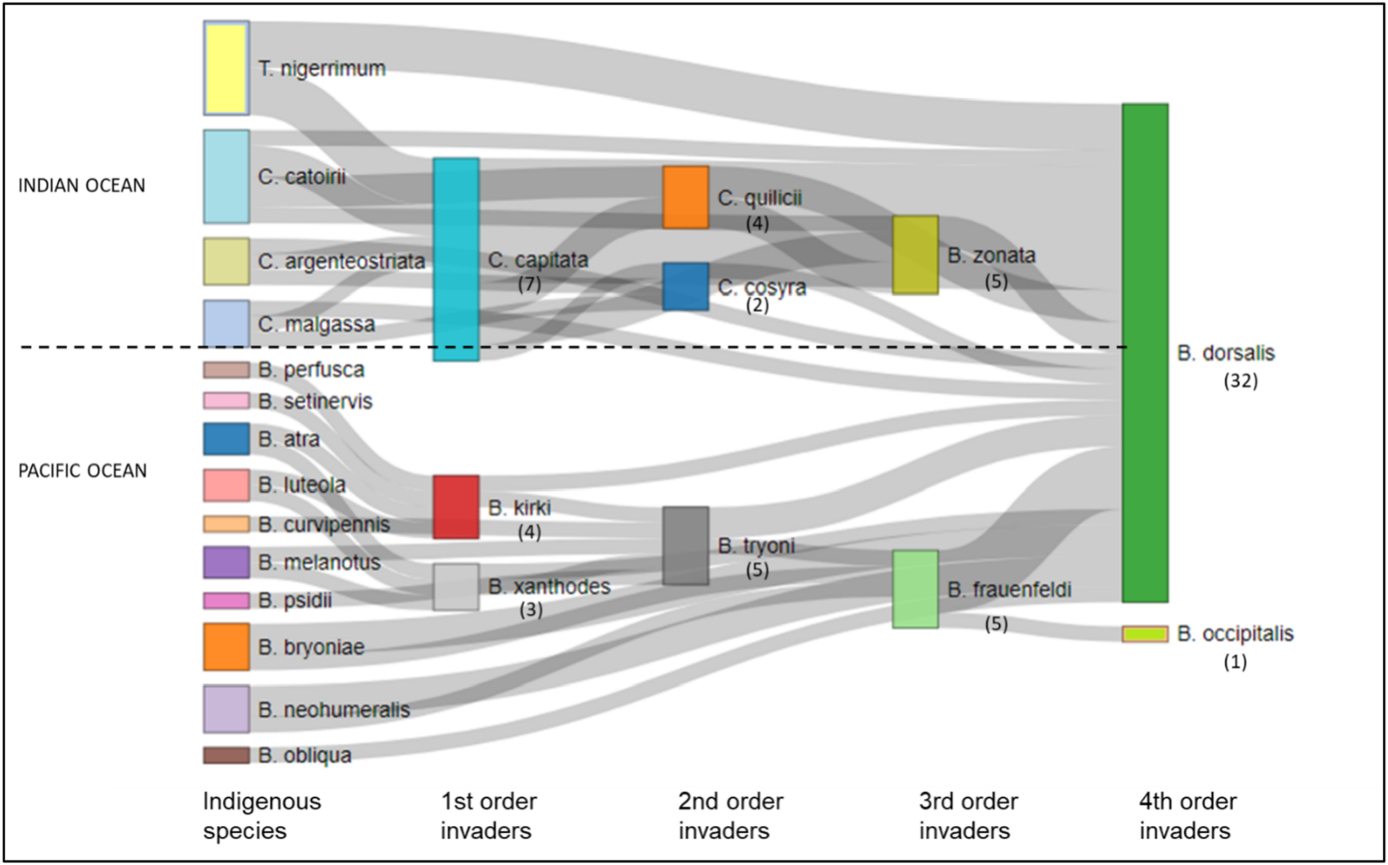
Diagram of the invasion links among polyphagous tephritids from Appendix S1. Each link represents a successful invasion by one species in the presence of another species. The number in brackets for each invasive species represents the number of invasions observed in the presence of another polyphagous species

These observations confirm previous hypotheses of directional invasions (Duyck et al., 2004). For some islands, we observed a series of invasions, i.e., the sequential invasion of the same island by several species. This is particularly relevant in La Réunion, Mauritius, and French Polynesia where four invaders have established in a sequential manner. From the invasion series reported, *B*. *dorsalis* was always the last species to spread when it is present. *Bactrocera dorsalis* invaded 13 islands where other polyphagous tephritid species were already present; the first case was in Hawaii in 1945, and the last one was in La Réunion in 2017. For *B*. *dorsalis*, this represents 17 and 15 invasion links, respectively, in Southwest Indian Ocean islands and Pacific islands (Figure 3, Appendix S1).

### 3.2 Causes of observed pattern: Interspecific competition hypothesis

The observed patterns could have several causes. First, the limitation of resident species by interspecific competition along with an increasing competitive ability of the next invaders can lead to directional links of invasions (Duyck et al., 2004, 2006). Although abundance data for the different tephritid species are not always available before and after invasions, some competitive displacements have been clearly documented. In La Réunion and in French Polynesia, *B*. *dorsalis* reduced the abundance and displaced dominant tephritids that were already present (Moquet et al., 2021; Vargas et al., 2012). Competitive displacements have also been well described on the African mainland where *B*. *dorsalis* has displaced four species *C*. *rosa*, *C*. *cosyra*, *C*. *quilicii*, and *C*. *capitata* in different countries (Ekesi et al., 2009; Mwatawala et al., 2009; Rwomushana et al., 2008). The importance of interspecific competition among polyphagous tephritids has been studied in detail in the laboratory with reared populations (Duyck et al., 2006); the results showed that each successive invader was a better competitor than the previous invaders or native species in terms of exploitative competition between larvae or of female interference. These hierarchies in competitive ability also follow an *r*/*K* continuum with the last species established being competitively dominant and more *K*‐selected than the already resident species (Duyck et al., 2007). Recent invaders, such as *B*. *dorsalis* or *B*. *zonata* tend to produce fewer, but larger, juveniles, delay the onset but increase the duration of reproduction, and survive longer than earlier ones (Duyck et al., 2007; Vargas et al., 2000).

Should *B*. *dorsalis* be considered a super invader? In most invasion series involving polyphagous tephritids documented to date, *B*. *dorsalis* appears as the last species to establish and to be dominant in terms of abundance (Hassani et al., 2016; Moquet et al., 2021; Vargas et al., 2012). This seems not to be the case, however, in Papua New Guinea (PNG) or in Madagascar; although *B*. *dorsalis* has invaded both places, the indigenous *B*. *frauenfeldi* remains very abundant on many hosts in PNG (Leblanc et al., 2013; Putulan et al., 2004), and the same is true for *C*. *malgassa* in Madagascar (Rasolofoarivao et al., 2021). These situations could be explained by ecological effects, such as a high diversity of fruit fly species in both places, or by evolutionary effects, such as the presence of different *B*. *dorsalis* strains (Schutze et al., 2015).

On the other hand, not all researchers agree that interspecific competition dominates relationships among Tephritidae. Tephritid abundance may not be generally limited by interspecific competition for fruit resources (Clarke, 2017), although abundances may be sufficiently high to result in interspecific competition during biological invasions. Also, in a recent study using joint species distribution modeling and network inference, Facon et al. (2021) suggest that interspecific competition among the tephritids of La Réunion was less important than expected. Interspecific competition may not be currently important, however, if niche partitioning caused by intense interspecific competition previously occurred; this paradox has been termed “the ghost of competition past” (Connell, 1980). Competition experiments involving the modification of species densities in the field are difficult (Hart et al., 2018) to perform but would help clarify the importance of interspecific competition when invasion occurs.

### 3.3 Causes of observed pattern: History, routes, and flows of commercial exchanges

Patterns of invasion are largely governed by global trade networks (Chapman et al., 2017). The observed patterns of sequential invasions by polyphagous Tephritidae may also have been caused by the history, routes, and flow of commercial exchanges. Overall, the invasion of Indian Ocean islands by polyphagous *Ceratitis* spp. from Africa preceded invasion by polyphagous *Bactrocera* spp. from Asia (Appendix S1). The increasing trade between Asia and the rest of the world has certainly promoted invasions by *Bactrocera* spp. Worldwide, several waves of invasions may have therefore led to this hierarchical mode of invasion, i.e., to directional invasions (Bertelsmeier & Ollier, 2021; Bonnamour et al., 2021). In the case of Tephritidae, the first wave with several *Ceratitis* spp. native to Africa may have spread across Indian Ocean islands, establishing “bridgeheads” from island to island according to the trade routes during the first part of the 20th century. Distance to the native area seems also to influence the probability of introduction: except for the invasion of Hawaii by *C*. *capitata*, *Ceratitis* spp. has not invaded the Pacific islands. In the late part of the 20th century, the second wave of *Bactrocera* spp. mostly native to Asia colonized most of the studied islands; this second wave was associated with new trade routes. Most recently, these islands have been invaded by *B*. *dorsalis* (Appendix S1).

### 3.4 Causes of the observed pattern: The bridgehead effect

As an increasing number of species are invasive worldwide, introduced populations often become a source of new invasions *via* secondary introductions, a pattern that has been termed the “bridgehead effect” (Bertelsmeier & Keller, 2018; Bertelsmeier & Ollier, 2021). The bridgehead effect may explain at least some cases of the observed pattern of sequential invasions by Tephritidae. As indicated by population genetics data, for instance, invasions of French Polynesia by *B*. *tryoni* involved invasive populations from New Caledonia (Popa‐Báez et al., 2020). In invading most of the Indian Ocean islands, *B*. *dorsalis* has probably moved from one island (one bridgehead) to another. The bridgehead effect may also be involved in the introduction of *B*. *dorsalis* in French Polynesia from an invasive population in Hawaii. Partial invasion routes are known for some species, but additional population genetics data will increase our understanding of the relevance of the bridgehead effect to the observed pattern of sequential invasions. Population genetics studies may also reveal that invasiveness may differ among populations or strains of tephritids (Clarke et al., 2005; Godefroid et al., 2015). Evolutionary adaptation *via* genetic admixture among several invasion events may also complete the bridgehead effect and lead to invasion success, which has been shown in the worldwide invasion of *Drosophila suzukii* (Fraimout et al., 2017).

### 3.5 Conclusion: Interactions among the different factors

We have shown that all cases of invasions by polyphagous Tephritidae in islands of the Pacific and the Southwest Indian Oceans are directional with the last species established being dominant. These sequential species establishments are driven by the interspecific competition ability among resident and exotic species but are also influenced by the history, routes, and flow of commercial exchanges and by the bridgehead effect. We have argued that the observed pattern is probably due to interaction among these different factors. The invasion of French Polynesia by *B*. *dorsalis*, for example, might be explained by *B*. *dorsalis* being more competitive than the resident species, but also because of the availability of a probable pathway for invasion (i.e., direct airplane transport of infested fruit between Hawaii and French Polynesia) and the presence of an invasive *B*. *dorsalis* population in Hawaii that was already adapted to the environment and resources in French Polynesia. Because many fruits and fruit plants have been spread by humans for many years, mango, guava, Indian almond, and other tephritid host plants are present throughout the tropics. That *Ceratitis* spp. has not invaded Pacific Ocean islands (except Hawaii) can be explained by the distance to the native area but also by the presence of more competitive *Bactrocera* spp. in most of the Pacific islands. Interestingly, Hawaii is one of the only Pacific islands where no endemic polyphagous *Bactrocera* species is present and is the only Pacific island where *C*. *capitata* has been able to establish a bridgehead and thrive (Vargas et al., 1995).

Our results indicate that islands that have not had an invasion series (or that have only 1st‐order invaders; see Figure 1 for an explanation of 1st‐order invaders) and that are well connected to areas hosting higher order invaders are especially susceptible to invasion by new species. This is the case in New Caledonia and Vanuatu, where *B*. *dorsalis* has not yet been established even though New Caledonia and Vanuatu are near areas where *B*. *dorsalis* is well established. We also argue that weakly competitive species, such as *C*. *capitata* (Duyck et al., 2006), have very little chance of invading islands where competitive species (such as *B*. *dorsalis*) already occupy most of the niches. Where the two species are present, however, the coexistence of *C*. *capitata* is possible only on refuge niches consisting of a few hosts like chili and coffee on which *B*. *dorsalis* does not develop.

The relationship between interspecific competition and overlap in the resource is complex but has important consequences for population dynamics and thus for the management of these species. Most important resources (in terms of quality and quantity) are generally shared by the polyphagous species (Charlery de la Masselière, Ravigné, et al., 2017). These species such as guava, Indian almond, or mango are present on most islands. While competition strength at the island scale is mediated by these plant species, other host plants not shared or preferred by all Tephritidae species may act as refuge niche and avoid generally complete extinction (Charlery de la Masselière, Ravigné, et al., 2017).

The question arises “Why are there no reciprocal invasions?” The opportunity to invade depends on the availability of a particular habitat with associated resources (a *filter niche*, David et al., 2017) where the introduced species can establish a viable population and resist competition before spreading to other habitats and resources. For polyphagous tephritids, this corresponds to warm and largely human‐modified lowlands where abundant and rich resources such as mango, guava, and Indian almond are present. It follows that, although *C*. *capitata* may be present in refuge niches in highland areas on particular hosts, there is very little chance that an invasion in these ecological niches could succeed.

### 3.6 Biosecurity applications

The information provided in this report could be used to increase biosecurity by encouraging those responsible for such security to do the following: define priorities in invasion risk; determine the presence of the host plant species in the area susceptible to invasion; determine the presence of competitive species in the connected areas; and identify the important pathways and routes (trade, flights) between the different areas. This information could also help biosecurity officers improve the network of traps used to monitor tephritid movement (Suckling et al., 2016). Regardless of the mechanisms involved in the invasion processes, the information presented here regarding invasion direction can be used for biosecurity. To ensure a better assessment of future threats, those who perform risk analysis should also pay attention to the resident fruit fly communities in countries that commercially produce the fruit in question in order to determine which commodities are threatened by tephritid invasion. For the sustainability of small islands, local fruit production must be promoted so as to avoid fruit imports according to the risk of introducing an invasive tephritid, especially when quarantine means are limited.

### 3.7 Perspectives

The observed pattern of sequential invasions therefore involves an interaction between species traits and human‐mediated dispersal, with the importance of these factors varying among cases. The different components of these factors and their interactions warrant additional study. For example, comparative analyses of interspecific interactions before and after invasion events are necessary to determine how invasive species affect the ecological network (Frost et al., 2019). Although researchers have recently followed changes in relationships between species and host range associated with changes in tephritid food webs (Moquet et al., 2021), these analyses should be expanded to additional cases so as to determine which patterns are general for different species and areas. Also needed are studies of life‐history traits of tephritids on numerous resources in the laboratory; the results of such studies can be used to determine fundamental niches, to predict distributions and abundance of tephritid populations in the field, and to better understand and estimate interspecific interactions (Facon et al., 2021). This work has been done for some of the studied species (Charlery de la Masselière, Facon, et al., 2017; Facon et al., 2021; Hafsi, Facon, et al., 2016), but it should be very informative to compare realized and fundamental niches at a larger scale including numerous islands and fruit fly species.

Additional analyses of human‐mediated dispersal (Chapman et al., 2017), known invasive routes, and the presence of invasive and resident species in many locations would improve our ability to identify important interactions among these factors. Tephritids of Pacific and Indian Ocean islands would be a good model system for such studies, which would complement and help provide a synthesis of existing data. Such studies should also consider climate change, because species may differ in their responses to changes in climate (Zhang et al., 2022), which may affect the observed hierarchy among species.

Finally, fruit fly management relies on strong international phytosanitary measures to manage established species and to avoid new species invasions (Phillips, 2013; Stephenson et al., 2003). If despite all biosecurity and phytosanitary measures fail to avoid an invasion, it is essential to improve all the panels of fruit fly management system, i.e., sterile insect technique (SIT), attract and kill systems, post‐harvest treatments, and the biological control (Bhoyroo et al., 2021; Follett & Neven, 2006; Garcia et al., 2020; Hafsi, Abbes, et al., 2016a; Suckling, 2003). Most of these management techniques are specific at the species level and therefore should consider, not only the competitively dominant species, but also other species that could have their population increased by lower populations of previously dominant species. More than ever, the fruit fly management system needs to be global focusing on all the fruit fly community present and regarding interactions among the biosecurity services of the different countries. Also, awareness of citizens should be enforced to prevent introduction with unconsidered behavior of bringing back fruit from travels, but also the involvement and the support of the community should be promoted in the context of incursion in a new area to complement fly management technical strategies (Ram, 2021).

## CONFLICT OF INTEREST

All the authors declare there is no competing interest related to the material of this manuscript.

## AUTHOR CONTRIBUTIONS

**Pierre‐François Duyck:** Conceptualization (lead); Formal analysis (lead); Investigation (lead); Methodology (lead); Visualization (lead); Writing – original draft (lead). **Christian Mille:** Conceptualization (supporting); Validation (supporting); Writing – review & editing (equal). **Hervé Jourdan:** Conceptualization (supporting); Validation (supporting); Writing – review & editing (equal).

## Supporting information

Appendix S1

## Data Availability

This work is a review of the findings published in the field of fruit fly invasions and not based on collecting experimental data. Data generated and analyzed in this study are provided in Appendix S1.

## References

[ece38880-bib-0001] Allaire, J. , Gandrud, C. , Kenton, R. , & Yetman, C. (2017). networkD3: D3 JavaScript Network Graphs from R. https://CRAN.R‐project.org/package=networkD3

[ece38880-bib-0002] Allwood, A. J. , Vueti, E. T. , Leblanc, L. , & Bull, R. (2002). Eradication of introduced Bactrocera species (Diptera: Tephritidae) in Nauru using male annihilation and protein bait application techniques. In Turning the tide: The eradication of invasive species. Proceedings of the International Conference on Eradication of Island Invasives (p. 19). IUCN Publications Services Unit.

[ece38880-bib-0003] Bertelsmeier, C. , & Keller, L. (2018). Bridgehead effects and role of adaptive evolution in invasive populations. Trends in Ecology & Evolution, 33(7), 527–534. 10.1016/j.tree.2018.04.014 29764688

[ece38880-bib-0004] Bertelsmeier, C. , & Ollier, S. (2021). Bridgehead effects distort global flows of alien species. Diversity and Distributions, 27(11), 2180–2189. 10.1111/ddi.13388

[ece38880-bib-0005] Bhoyroo, R. D. , Facknath, S. , & Sookar, P. (2021). Life table of Bactrocera zonata (Saunders) (Diptera: Tephritidae) for Sterile Insect Technique (SIT) in Mauritius. African Entomology, 29(2), 361–369. 10.4001/003.029.0361

[ece38880-bib-0006] Bonnamour, A. , Gippet, J. M. W. , & Bertelsmeier, C. (2021). Insect and plant invasions follow two waves of globalisation. Ecology Letters, 24(11), 2418–2426. 10.1111/ele.13863 34420251 PMC9290749

[ece38880-bib-0007] Broennimann, O. , Treier, U. A. , Müller‐Schärer, H. , Thuiller, W. , Peterson, A. T. , & Guisan, A. (2007). Evidence of climatic niche shift during biological invasion. Ecology Letters, 10(8), 701–709. 10.1111/j.1461-0248.2007.01060.x 17594425

[ece38880-bib-0008] Chapman, D. , Purse, B. V. , Roy, H. E. , Bullock, J. (2017). Global trade networks determine the distribution of invasive non‐native species. Global Ecology and Biogeography, 26(8), 907–917. 10.1111/geb.12599

[ece38880-bib-0009] Charlery de la Masselière, M. , Facon, B. , Hafsi, A. , & Duyck, P.‐F. (2017). Diet breadth modulates preference—Performance relationships in a phytophagous insect community. Scientific Reports, 7(1), 16934. 10.1038/s41598-017-17231-2 29208939 PMC5717236

[ece38880-bib-0010] Charlery de la Masselière, M. , Ravigné, V. , Facon, B. , Lefeuvre, P. , Massol, F. , Quilici, S. , & Duyck, P.‐F. (2017). Changes in phytophagous insect host ranges following the invasion of their community: Long‐term data for fruit flies. Ecology and Evolution, 7(14), 5181–5190. 10.1002/ece3.2968 28770058 PMC5528217

[ece38880-bib-0011] Clarke, A. R. (2017). Why so many polyphagous fruit flies (Diptera: Tephritidae)? A further contribution to the ‘generalism’ debate. Biological Journal of the Linnean Society, 120(2), 245–257. 10.1111/bij.12880

[ece38880-bib-0012] Clarke, A. R. , Armstrong, K. F. , Carmichael, A. E. , Milne, J. R. , Raghu, S. , Roderick, G. K. , & Yeates, D. K. (2005). Invasive phytophagous pests arising through a recent tropical evolutionary radiation: The *Bactrocera dorsalis* complex of fruit flies. Annual Review of Entomology, 50, 293–319. 10.1146/annurev.ento.50.071803.130428 15355242

[ece38880-bib-0013] Connell, J. H. (1980). Diversity and the coevolution of competitors, or the ghost of competition past. Oikos, 35(2), 131. 10.2307/3544421

[ece38880-bib-0014] David, P. , Thébault, E. , Anneville, O. , Duyck, P.‐F. , Chapuis, E. , & Loeuille, N. (2017). Impacts of invasive species on food webs. Advances in Ecological Research, 56, 1–60. 10.1016/bs.aecr.2016.10.001

[ece38880-bib-0015] De Meyer, M. , Quilici, S. , Franck, A. , Chadhouliati, A. C. , Issimaila, M. A. , Youssoufa, M. A. , Abdoul‐Karime, A.‐L. , Barbet, A. , Attié, M. , & White, I. M. (2012). Records of frugivorous fruit flies (Diptera: Tephritidae: Dacini) from the Comoro archipelago. African Invertebrates, 53(1), 69–67. 10.5733/afin.053.0104

[ece38880-bib-0016] Duyck, P.‐F. , David, P. , Junod, G. , Brunel, C. , Dupont, R. , & Quilici, S. (2006). Importance of competition mechanisms in successive invasions by polyphagous tephritids in La Réunion Island. Ecology, 87(7), 1770–1780. 10.1890/0012-9658(2006)87#;1770:IOCMIS#;2.0.CO;2 16922326

[ece38880-bib-0017] Duyck, P.‐F. , David, P. , & Quilici, S. (2004). A review of relationships between interspecific competition and invasions in fruit flies (Diptera: Tephritidae). Ecological Entomology, 29(5), 511–520. 10.1111/j.0307-6946.2004.00638.x

[ece38880-bib-0018] Duyck, P.‐F. , David, P. , & Quilici, S. (2007). Can more K‐selected species be better invaders? A case study of fruit flies in La Réunion: r‐K trade‐off in biological invasions. Diversity and Distributions, 13(5), 535–543. 10.1111/j.1472-4642.2007.00360.x

[ece38880-bib-0019] Ekesi, S. , Billah, M. K. , Nderitu, P. W. , Lux, S. A. , & Rwomushana, I. (2009). Evidence for competitive displacement of *Ceratitis cosyra* by the invasive fruit fly *Bactrocera invadens* (Diptera: Tephritidae) on mango and mechanisms contributing to the displacement. Journal of Economic Entomology, 102(3), 981–991. 19610411 10.1603/029.102.0317

[ece38880-bib-0020] Ekesi, S. , De Meyer, M. , Mohamed, S. A. , Virgilio, M. , & Borgemeister, C. (2016). Taxonomy, ecology and management of native and exotic fruit fly species in Africa. Annual Review of Entomology, 61, 219–238. 10.1146/annurev-ento-010715-023603 26735644

[ece38880-bib-0021] Facon, B. , Hafsi, A. , Charlery de la Masselière, M. , Robin, S. , Massol, F. , Dubart, M. , Chiquet, J. , Frago, E. , Chiroleu, F. , Duyck, P.‐F. , & Ravigné, V. (2021). Joint species distributions reveal the combined effects of host plants, abiotic factors and species competition as drivers of species abundances in fruit flies. Ecology Letters, 24(9), 1905–1916. 10.1111/ele.13825 34231296

[ece38880-bib-0022] Follett, P. A. , & Neven, L. G. (2006). Current trends in quarantine entomology. Annual Review of Entomology, 51(1), 359–385. 10.1146/annurev.ento.49.061802.123314 16332216

[ece38880-bib-0023] Fournier, A. , Penone, C. , Pennino, M. G. , & Courchamp, F. (2019). Predicting future invaders and future invasions. Proceedings of the National Academy of Sciences of the United States of America, 116(16), 7905–7910. 10.1073/pnas.1803456116 30926662 PMC6475384

[ece38880-bib-0024] Fraimout, A. , Debat, V. , Fellous, S. , Hufbauer, R. A. , Foucaud, J. , Pudlo, P. , Marin, J.‐M. , Price, D. K. , Cattel, J. , Chen, X. , Deprá, M. , François Duyck, P. , Guedot, C. , Kenis, M. , Kimura, M. T. , Loeb, G. , Loiseau, A. , Martinez‐Sañudo, I. , Pascual, M. , … Estoup, A. (2017). Deciphering the routes of invasion of *Drosophila suzukii* by means of ABC random forest. Molecular Biology and Evolution, 34, 980–996. 10.1093/molbev/msx050 28122970 PMC5400373

[ece38880-bib-0025] Frost, C. M. , Allen, W. J. , Courchamp, F. , Jeschke, J. M. , Saul, W.‐C. , & Wardle, D. A. (2019). Using network theory to understand and predict biological invasions. Trends in Ecology & Evolution, 34(9), 831–843. 10.1016/j.tree.2019.04.012 31155422

[ece38880-bib-0026] Garcia, F. R. M. , Ovruski, S. M. , Suárez, L. , Cancino, J. , & Liburd, O. E. (2020). Biological control of tephritid fruit flies in the Americas and Hawaii: A review of the use of parasitoids and predators. Insects, 11(10), 662. 10.3390/insects11100662 PMC7600837 32993000

[ece38880-bib-0027] Godefroid, M. , Cruaud, A. , Rossi, J.‐P. , & Rasplus, J.‐Y. (2015). Assessing the risk of invasion by tephritid fruit flies: Intraspecific divergence matters. PLoS One, 10(8), e0135209. 10.1371/journal.pone.0135209 26274582 PMC4537207

[ece38880-bib-0028] Hafsi, A. , Abbes, K. , Harbi, A. , Duyck, P.‐F. , & Chermiti, B. (2016). Attract‐and‐kill systems efficiency against *Ceratitis capitata* (Diptera: Tephritidae) and effects on non‐target insects in peach orchards. Journal of Applied Entomology, 140(1–2), 28–36. 10.1111/jen.12259

[ece38880-bib-0029] Hafsi, A. , Facon, B. , Ravigné, V. , Chiroleu, F. , Quilici, S. , Chermiti, B. , & Duyck, P.‐F. (2016). Host plant range of a fruit fly community (Diptera: Tephritidae): Does fruit composition influence larval performance? BMC Ecology, 16, 40. 10.1186/s12898-016-0094-8 27650549 PMC5030732

[ece38880-bib-0030] Hart, S. P. , Freckleton, R. P. , & Levine, J. M. (2018). How to quantify competitive ability. Journal of Ecology, 106(5), 1902–1909. 10.1111/1365-2745.12954

[ece38880-bib-0031] Hassani, I. M. , Raveloson‐Ravaomanarivo, L. H. , Delatte, H. , Chiroleu, F. , Allibert, A. , Nouhou, S. , Quilici, S. , & Duyck, P. F. (2016). Invasion by *Bactrocera dorsalis* and niche partitioning among tephritid species in Comoros. Bulletin of Entomological Research, 6, 1–10. 10.1017/S0007485316000456 27312045

[ece38880-bib-0032] Hulme, P. E. (2010). Biosecurity: The changing face of invasion biology. In D. M. Richardson (Ed.), Fifty years of invasion ecology (pp. 301–314). Wiley‐Blackwell.

[ece38880-bib-0033] Hulme, P. E. (2021). Unwelcome exchange: International trade as a direct and indirect driver of biological invasions worldwide. One Earth, 4(5), 666–679. 10.1016/j.oneear.2021.04.015

[ece38880-bib-0034] Leblanc, L. , Vueti, E. T. , & Allwood, A. J. (2013). Host plant records for fruit flies (Diptera: Tephritidae: Dacini) in the Pacific islands: 2. Infestation Statistics on Economic Hosts. Proceedings of the Hawaiian Entomological Society, 45, 36.

[ece38880-bib-0035] MacLachlan, M. J. , Liebhold, A. M. , Yamanaka, T. , & Springborn, M. R. (2021). Hidden patterns of insect establishment risk revealed from two centuries of alien species discoveries. Science Advances, 7(44), eabj1012. 10.1126/sciadv.abj1012 34705509 PMC8550319

[ece38880-bib-0036] Moquet, L. , Payet, J. , Glenac, S. , & Delatte, H. (2021). Niche shift of tephritid species after the Oriental fruit fly (*Bactrocera dorsalis*) invasion in La Réunion. Diversity and Distributions, 27(1), 109–129. 10.1111/ddi.13172

[ece38880-bib-0037] Moser, D. , Lenzner, B. , Weigelt, P. , Dawson, W. , Kreft, H. , Pergl, J. , Pyšek, P. , van Kleunen, M. , Winter, M. , Capinha, C. , Cassey, P. , Dullinger, S. , Economo, E. P. , García‐Díaz, P. , Guénard, B. , Hofhansl, F. , Mang, T. , Seebens, H. , & Essl, F. (2018). Remoteness promotes biological invasions on islands worldwide. Proceedings of the National Academy of Sciences of the United States of America, 115(37), 9270–9275. 10.1073/pnas.1804179115 30158167 PMC6140508

[ece38880-bib-0038] Mwatawala, M. W. , De Meyer, M. , Makundi, R. H. , & Maerere, A. P. (2009). Host range and distribution of fruit‐infesting pestiferous fruit flies (Diptera, Tephritidae) in selected areas of Central Tanzania. Bulletin of Entomological Research, 99(06), 629–641. 10.1017/S0007485309006695 19323850

[ece38880-bib-0039] N'depo, O. R. , Hala, N. , N'da Adopo, A. , Coulibaly, F. , Kouassi, P. K. , Vayssieres, J. F. , & De Meyer, M. (2015). Effective chemical control of fruit flies (Diptera: Tephritidae) pests in mango orchards in northern Côte‐d’Ivoire. International Journal of Biological and Chemical Sciences, 9(3), 1299. 10.4314/ijbcs.v9i3.15

[ece38880-bib-0040] Paini, D. R. , Sheppard, A. W. , Cook, D. C. , De Barro, P. J. , Worner, S. P. , & Thomas, M. B. (2016). Global threat to agriculture from invasive species. Proceedings of the National Academy of Sciences of the United States of America, 113(27), 7575–7579. 10.1073/pnas.1602205113 27325781 PMC4941431

[ece38880-bib-0041] Phillips, C. (2013). Living without fruit flies: Biosecuring horticulture and its markets. Environment and Planning A: Economy and Space, 45(7), 1679–1694. 10.1068/a45274

[ece38880-bib-0042] Popa‐Báez, Á.‐D. , Catullo, R. , Lee, S. F. , Yeap, H. L. , Mourant, R. G. , Frommer, M. , Sved, J. A. , Cameron, E. C. , Edwards, O. R. , Taylor, P. W. , & Oakeshott, J. G. (2020). Genome‐wide patterns of differentiation over space and time in the Queensland fruit fly. Scientific Reports, 10(1), 10788. 10.1038/s41598-020-67397-5 32612249 PMC7329829

[ece38880-bib-0043] Putulan, D. , Sar, S. , Drew, R. , Raghu, S. , & Clarke, A. (2004). Fruit and vegetable movement on domestic flights in Papua New Guinea and the risk of spreading pest fruit‐flies (Diptera: Tephritidae). International Journal of Pest Management, 50(1), 17–22. 10.1080/09670870310001626329

[ece38880-bib-0044] Qin, Y. , Paini, D. R. , Wang, C. , Fang, Y. , & Li, Z. (2015). Global establishment risk of economically important fruit fly species (Tephritidae). PLoS One, 10(1), e0116424. 10.1371/journal.pone.0116424 25588025 PMC4294639

[ece38880-bib-0045] R Core Team (2021). R: A language and environment for statistical computing. R Foundation for Statistical Computing.

[ece38880-bib-0046] Ram, R. (2021). Community responses to biosecurity regulations during a biosecurity outbreak: An Auckland, New Zealand case study. Community Development, 52(1), 42–60. 10.1080/15575330.2020.1831564

[ece38880-bib-0047] Rasolofoarivao, H. , Raveloson Ravaomanarivo, L. H. , & Delatte, H. (2021). Host plant ranges of fruit flies (Diptera: Tephritidae) in Madagascar. Bulletin of Entomological Research, 112(1), 1–12. 10.1017/S0007485321000511 35225174

[ece38880-bib-0064] Russell, J. , Meyer, J. , Holmes, N. , & Pagad, S. (2017). Invasive alien species on islands: Impacts, distribution, interactions and management. Environmental Conservation, 44(4), 359–370. 10.1017/S0376892917000297

[ece38880-bib-0048] Rwomushana, I. , Ekesi, S. , Gordon, I. , & Ogol, C. K. (2008). Host plants and host plant preference studies for *Bactrocera invadens* (Diptera: Tephritidae) in Kenya, a new invasive fruit fly species in Africa. Annals of the Entomological Society of America, 101(2), 331–340. 10.1603/0013-8746(2008)101#;331:hpahpp#;2.0.co;2

[ece38880-bib-0049] Schutze, M. K. , Aketarawong, N. , Amornsak, W. , Armstrong, K. F. , Augustinos, A. A. , Barr, N. , Bo, W. , Bourtzis, K. , Boykin, L. M. , & Caceres, C. (2015). Synonymization of key pest species within the *Bactrocera dorsalis* species complex (Diptera: Tephritidae): Taxonomic changes based on a review of 20 years of integrative morphological, molecular, cytogenetic, behavioural and chemoecological data. Systematic Entomology, 40(2), 456–471.

[ece38880-bib-0050] Seebens, H. , Blackburn, T. M. , Dyer, E. E. , Genovesi, P. , Hulme, P. E. , Jeschke, J. M. , Pagad, S. , Pyšek, P. , van Kleunen, M. , Winter, M. , Ansong, M. , Arianoutsou, M. , Bacher, S. , Blasius, B. , Brockerhoff, E. G. , Brundu, G. , Capinha, C. , Causton, C. E. , Celesti‐Grapow, L. , … Essl, F. (2018). Global rise in emerging alien species results from increased accessibility of new source pools. Proceedings of the National Academy of Sciences of the United States of America, 115(10), E2264–E2273. 10.1073/pnas.1719429115 29432147 PMC5877962

[ece38880-bib-0051] Seebens, H. , Blackburn, T. M. , Dyer, E. E. , Genovesi, P. , Hulme, P. E. , Jeschke, J. M. , Pagad, S. , Pyšek, P. , Winter, M. , Arianoutsou, M. , Bacher, S. , Blasius, B. , Brundu, G. , Capinha, C. , Celesti‐Grapow, L. , Dawson, W. , Dullinger, S. , Fuentes, N. , Jäger, H. , … Essl, F. (2017). No saturation in the accumulation of alien species worldwide. Nature Communications, 8(1), 14435. 10.1038/ncomms14435 PMC5316856 28198420

[ece38880-bib-0052] Sikes, B. A. , Bufford, J. L. , Hulme, P. E. , Cooper, J. A. , Johnston, P. R. , & Duncan, R. P. (2018). Import volumes and biosecurity interventions shape the arrival rate of fungal pathogens. PLoS Biology, 16(5), e2006025. 10.1371/journal.pbio.2006025 29851948 PMC5978781

[ece38880-bib-0053] Simberloff, D. , Martin, J.‐L. , Genovesi, P. , Maris, V. , Wardle, D. A. , Aronson, J. , Courchamp, F. , Galil, B. , García‐Berthou, E. , Pascal, M. , Pyšek, P. , Sousa, R. , Tabacchi, E. , & Vilà, M. (2013). Impacts of biological invasions: What’s what and the way forward. Trends in Ecology & Evolution, 28(1), 58–66. 10.1016/j.tree.2012.07.013 22889499

[ece38880-bib-0054] Stephenson, B. P. , Gill, G. S. C. , Randall, J. L. , & Wilson, J. A. (2003). Biosecurity approaches to surveillance and response for new plant pest species. New Zealand Plant Protection, 56, 5–9. 10.30843/nzpp.2003.56.6023

[ece38880-bib-0055] Suckling, D. M. (2003). Applying the sterile insect technique for biosecurity benefits and constraints. New Zealand Plant Protection, 56, 21–26. 10.30843/nzpp.2003.56.6026

[ece38880-bib-0056] Suckling, D. M. , Kean, J. M. , Stringer, L. D. , Cáceres‐Barrios, C. , Hendrichs, J. , Reyes‐Flores, J. , & Dominiak, B. C. (2016). Eradication of tephritid fruit fly pest populations: Outcomes and prospects: Eradication of tephritid fruit fly pest populations. Pest Management Science, 72(3), 456–465. 10.1002/ps.3905 25204807

[ece38880-bib-0057] Vargas, R. I. , Leblanc, L. , Putoa, R. , & Piñero, J. C. (2012). Population dynamics of three Bactrocera spp. fruit flies (Diptera: Tephritidae) and two introduced natural enemies, Fopius arisanus (Sonan) and Diachasmimorpha longicaudata (Ashmead) (Hymenoptera: Braconidae), after an invasion by Bactrocera dorsalis (Hendel) in Tahiti. Biological Control, 60(2), 199–206. 10.1016/j.biocontrol.2011.10.012

[ece38880-bib-0058] Vargas, R. I. , Piñero, J. , & Leblanc, L. (2015). An overview of pest species of Bactrocera fruit flies (Diptera: Tephritidae) and the integration of biopesticides with other biological approaches for their management with a focus on the Pacific region. Insects, 6(2), 297–318. 10.3390/insects6020297 26463186 PMC4553480

[ece38880-bib-0059] Vargas, R. I. , Walsh, W. A. , Kanehisa, D. , Stark, J. D. , & Nishida, T. (2000). Comparative demography of three Hawaiian fruit flies (Diptera: Tephritidae) at alternating temperatures. Annals of the Entomological Society of America, 93(1), 75–81. 10.1603/0013-8746(2000)093#;0075:cdothf#;2.0.co;2

[ece38880-bib-0060] Vargas, R. I. , Walsh, W. A. , & Nishida, T. (1995). Colonization of newly planted coffee fields: Dominance of Mediterranean fruit fly over Oriental fruit fly (Diptera: Tephritidae). Journal of Economic Entomology, 88(3), 620–627. 10.1093/jee/88.3.620

[ece38880-bib-0061] White, E. M. , Wilson, J. C. , & Clarke, A. R. (2006). Biotic indirect effects: A neglected concept in invasion biology. Diversity and Distributions, 12(4), 443–455. 10.1111/j.1366-9516.2006.00265.x

[ece38880-bib-0062] White, I. M. , & Elson‐Harris, M. M. (1992). Fruit flies of economic significance: Their identification and bionomics. CAB International.

[ece38880-bib-0063] Zhang, Y. , Hughes, A. C. , Zhao, Z. , Li, Z. , & Qin, Y. (2022). Including climate change to predict the global suitable area of an invasive pest: Bactrocera correcta (Diptera: Tephritidae). Global Ecology and Conservation, 34, e02021. 10.1016/j.gecco.2022.e02021

