# Supplementary material for: Sequential invasions by fruit flies (Diptera: Tephritidae) in Pacific and Indian Ocean islands: A systematic review
Source: Ecol Evol. 2022 Apr 30;12(5):e8880. doi: 10.1002/ece3.8880 (PMC9055289; doi:10.1002/ece3.8880)
Supplement: Supplementary file 1 — Appendix S1 [file ECE3-12-e8880-s001.docx]

**Appendix S1.** Cases of polyphagous tephritid species that have been introduced to Pacific and Southwest Indian Ocean Islands where other polyphagous species of the family were pre-established.

|  |  |  |  |  |
| --- | --- | --- | --- | --- |
| **Invasive species** | **Pre established species** | **Invaded area** | **Invasion date**  **or period** | **References** |
|  |  |  |  |  |
| *B. dorsalis* | *B. bryoniae* | PNG | 1992 | 13; 18 |
|  |  | Australia | 1995 | 8 |
|  | *B. frauenfeldi* | PNG | 1992 | 13; 18 |
|  |  | Nauru | 1985 | 2; 23 |
|  |  | Palau | 1996 | 22 |
|  |  | Australia | 1995 | 8 |
|  | *B. kirki* | French Polynesia | 1996 | 1; 12 |
|  | *B. neohumeralis* | PNG | 1992 | 13; 18 |
|  |  | Australia | 1995 | 8 |
|  | *B. obliqua* | PNG | 1992 | 13; 18 |
|  | *B. tryoni* | French Polynesia | 1996 | 1; 12 |
|  |  | Australia | 1995 | 8 |
|  | *B. xanthodes* | French Polynesia | 1996 | 1; 12 |
|  | *B. zonata* | Reunion | 2017 | 16 |
|  |  | Mauritius | 1996 | 14 |
|  | *C. argenteostriata* | Madagascar | 2010 | 19 |
|  | *C. capitata* | Madagascar | 2010 | 19 |
|  |  | Hawaii | 1945 | 11; 20 |
|  |  | Reunion | 2017 | 16 |
|  |  | Mauritius | 1996 | 14 |
|  |  | Grande-Comore | 2005 | 5; 10 |
|  |  | Anjouan | after 2005 | 5; 10 |
|  |  | Moheli | after 2005 | 5; 10 |
|  |  | Mayotte | 2007 | 4 |
|  | *C. catoirii* | Reunion | 2017 | 16 |
|  | *C. cosyra* | Madagascar | 2010 | 19 |
|  | *C. malgassa* | Madagascar | 2010 | 19 |
|  | *C. quilicii* | Reunion | 2017 | 16 |
|  |  | Mauritius | 1996 | 14 |
|  | *T. nigerrimum* | Grande-Comore | 2005 | 5; 10 |
|  |  | Anjouan | after 2005 | 5; 10 |
|  |  | Moheli | after 2005 | 5; 10 |
| *B. frauenfeldi* | *B. bryoniae* | Australia | 1974 | 21 |
|  | *B. neohumeralis* | Australia | 1974 | 21 |
|  | *B. tryoni* | Australia | 1974 | 21 |
| *B. kirki* | *B. atra* | French Polynesia | 1928 | 12 |
|  | *B. luteola* | French Polynesia | 1928 | 12 |
|  | *B. perfusca* | French Polynesia | 1928 | 12 |
|  | *B. setinervis* | French Polynesia | 1928 | 12 |
| *B. occipitalis* | *B. frauenfeldi* | Palau | 1996 | 22 |
| *B. tryoni* | *B. curvipennis* | New Caledonia | 1969 | 15 |
|  | *B. kirki* | French Polynesia | 1970 | 12 |
|  | *B. luteola* | French Polynesia | 1970 | 12 |
|  | *B. psidii* | New Caledonia | 1969 | 15 |
|  | *B. xanthodes* | French Polynesia | 1970 | 12 |
| *B. xanthodes* | *B. atra* | French Polynesia | 1998 | 12 |
|  | *B. luteola* | French Polynesia | 1998 | 12 |
|  | *B. melanotus* | Cook | 1970 | 1 |
| *B. zonata* | *C. capitata* | Reunion | 1991 | 3 |
|  |  | Mauritius | 1987 | 24 |
|  | *C. catoirii* | Reunion | 1991 | 3 |
|  | *C. quilicii* | Reunion | 1991 | 3 |
|  |  | Mauritius | 1987 | 24 |
| *C. capitata* | *C. argenteostriata* | Madagascar | 1962 | 6 |
|  | *C. catoirii* | Reunion | 1955 | 7 |
|  |  | Mauritius | 1953 | 17 |
|  | *C. malgassa* | Madagascar | 1962 | 6 |
| *C. cosyra* | *C. argenteostriata* | Madagascar | 1965-1984 | 9 |
|  | *C. capitata* | Madagascar | 1965-1984 | 9 |
|  | *C. malgassa* | Madagascar | 1965-1984 | 9 |
| *C. quilicii* | *C. capitata* | Reunion | 1939 | 7 |
|  |  | Mauritius | 1942 | 17 |
|  | *C. catoirii* | Reunion | 1939 | 7 |
|  |  | Mauritius | 1942 | 17 |
|  |  |  |  |  |
|  |  |  |  |  |

**References**

1. Allwood, A. J., & Drew, R. A. I. (1997). *Management of fruit flies in the Pacific. A regional symposium*. Nadi, Fiji 28-31 October 1996. ACIAR Proceedings No 76. 267pp.

2. Allwood, A. J., Vueti, E. T., Leblanc, L., & Bull, R. (2002). Eradication of introduced *Bactrocera* species (Diptera: Tephritidae) in Nauru using male annihilation and protein bait application techniques. In *Turning the tide: the eradication of invasive species. Proceedings of the International Conference on Eradication of Island Invasives* (pp. 19). Cambridge, U.K.: IUCN Publications Services Unit.

3. Charlery de la Masselière, M., Ravigné, V., Facon, B., Lefeuvre, P., Massol, F., Quilici, S., & Duyck, P. F. (2017). Changes in phytophagous insect host ranges following the invasion of their community: Long‐term data for fruit flies. *Ecology and Evolution, 7*(14), 5181-5190. doi:10.1002/ece3.2968

4. De Meyer, M., Copeland, R. S., Lux, S. A., Mansell, M., Quilici, S., Wharton, R., . . . Zenz, N. J. (2002). *Annotated check list of host plants for Afrotropical fruit flies (Diptera: Tephritidae) of the genus Ceratitis*. Retrieved from Tervuren, Belgium:

5. De Meyer, M., Quilici, S., Franck, A., Chadhouliati, A. C., Issimaila, M. A., Youssoufa, M. A., . . . White, I. M. (2012). Records of frugivorous fruit flies (Diptera: Tephritidae: Dacini) from the Comoro archipelago. *African Invertebrates, 53*(1), 69-67.

6. Dubois, J. (1965). La mouche des fruits malgache (*Ceratitis malagassa* Munro) et autres insectes des agrumes, pêchers et pruniers à Madagascar. *Fruits, 20*(9), 435-460.

7. Etienne, J. (1972). Les principales Trypétides nuisibles de l'île de La Réunion. *Annales de la Societé Entomologique de France, 8*(2), 485-491.

8. Fay, H. A., Drew, R. A. I., & Lloyd, A. C. (1997). Eradication program for papaya fruit fly (*Bactrocera papayae* Drew and Hancock) in north Queensland. In A. J. Allwood & R. A. I. Drew (Eds.), *Management of fruit flies in the Pacific : a regional symposium* (Vol. 76, pp. 259-261 ): ACIAR Proeedings.

9. Hancock, D. L. (1984). Ceratitinae (Diptera:Tephritidae) from the Malagasy subregion. *Journal of the Entomological Society of Southern Africa, 2*, 277-301.

10. Hassani, I. M., Raveloson-Ravaomanarivo, L. H., Delatte, H., Chiroleu, F., Allibert, A., Nouhou, S., . . . Duyck, P. F. (2016). Invasion by *Bactrocera dorsalis* and niche partitioning among tephritid species in Comoros. *Bulletin of Entomological Research, 6*, 1-10. doi:doi:10.1017/S0007485316000456

11. Keiser, I., Kobayashi, R. M., Miyashita, D. H., Harris, E. J., Schneider, E. J., & Chambers, D. L. (1974). Suppression of Mediterranean fruit flies by Oriental fruit flies in mixed infestations in guava. *Journal of Economic Entomology, 67*(3), 355-360.

12. Leblanc, L., & Putoa, R. (2000). Les mouches des fruits de Polynésie Française et des Îles Pitcairn. *Fiche technique no. 29 du Service de la Protection des Végétaux, Secrétariat Général de la communauté du Pacifique. Nouméa, Nouvelle-Calédonie*.

13. Leblanc, L., Vueti, E. T., & Allwood, A. J. (2013). Host Plant Records for Fruit Flies (Diptera: Tephritidae: Dacini) in the Pacific Islands: 2. Infestation Statistics on Economic Hosts. *Proceedings of the Hawaiian Entomological Society, 45*, 36.

14. Mauremootoo, J. R., Pandoo, S., Bachraz, V., Buldowoo, I., & Cole, N. C. (2019). Invasive species management in Mauritius : From the reactive to the proactive–the National Invasive Species Management Strategy and its implementation. In M. N. C. C. R. Veitch, A. R. Martin, J. C. Russell, & C. J. West (Ed.), *Island invasives: Scaling up to meet the challenge. Proceedings of the international conference on island invasives 2017* (pp. 503-509): IUCN, International Union for Conservation of Nature.

15. Mille, C. (2010). *Les mouches des fruits de Nouvelle-Calédonie (Diptera, Tephritidae) : systématique, comportement, dynamique et gestion des populations.* (PhD), Université de la Nouvelle-Calédonie.,

16. Moquet, L., Payet, J., Glenac, S., & Delatte, H. (2021). Niche shift of tephritid species after the Oriental fruit fly ( *Bactrocera dorsalis* ) invasion in La Réunion. *Diversity and Distributions, 27*(1), 109-129. doi:10.1111/ddi.13172

17. Orian, A. J. E., & Moutia, L. A. (1960). Fruit flies (Trypetidae) of economic importance in Mauritius. *Revue Agricole et Sucrière de l'Ile Maurice, 39*, 142-150.

18. Putulan, D., Sar, S., Drew, R., Raghu, S., & Clarke, A. (2004). Fruit and vegetable movement on domestic flights in Papua New Guinea and the risk of spreading pest fruit-flies (Diptera: Tephritidae). *International Journal of Pest Management, 50*(1), 17-22. doi:10.1080/09670870310001626329

19. Rasolofoarivao, H., Raveloson Ravaomanarivo, L. H., & Delatte, H. (2021). Host plant ranges of fruit flies (Diptera: Tephritidae) in Madagascar. *Bulletin of Entomological Research*, 1-12. doi:10.1017/S0007485321000511

20. Reitz, S. R., & Trumble, J. T. (2002). Competitive displacement among insects and arachnids. *Annual Review of Entomology, 47*, 435-465.

21. Royer, J. E., Wright, C. L., & Hancock, D. L. (2016). *Bactrocera frauenfeldi* (Diptera: Tephritidae), an invasive fruit fly in Australia that may have reached the extent of its spread due to environmental variables. *Austral Entomology, 55*(1), 100-111. doi:10.1111/aen.12155

22. Sengebau, F., Waqa, N., & Vueti, E. T. (2005). *Fruit flies in Palau*. Retrieved from

23. Suckling, D. M., Kean, J. M., Stringer, L. D., Cáceres-Barrios, C., Hendrichs, J., Reyes-Flores, J., & Dominiak, B. C. (2016). Eradication of tephritid fruit fly pest populations: outcomes and prospects: Eradication of tephritid fruit fly pest populations. *Pest Management Science, 72*(3), 456-465. doi:10.1002/ps.3905

24. White, I. M., De Meyer, M., & Stonehouse, J. M. (2000). A Review of native and introduced fruit flies (Diptera, Tephritidae) in the Indian Ocean islands of Mauritius, Réunion and Seychelles. In N. S. Price & S. I. Seewooruthun (Eds.), *Proceedings of the Indian Ocean Commission regional fruit fly symposium* (pp. 15-21). Flic en Flac, Mauritius: Indian Ocean Commission / European Union.
